# Transcription Factor Dynamics in Neuroendocrine Prostate Cancer Development

**DOI:** 10.1007/s44178-025-00197-x

**Published:** 2025-10-24

**Authors:** Yu Wang, Yuzhuo Wang

**Affiliations:** 1 Department of Urologic Sciences, Faculty of Medicine, University of British Columbia, Vancouver, BC Canada; 2 Vancouver Prostate Centre, Vancouver, BC Canada; 3 Department of Experimental Therapeutics, BC Cancer, Vancouver, BC Canada

**Keywords:** Prostate cancer, Neuroendocrine prostate cancer (NEPC), Transcription factors, Lineage plasticity, Tumor dormancy, Three-phase hypothesis

## Abstract

Treatment-induced neuroendocrine prostate cancer (NEPC) represents a lethal evolution of prostate adenocarcinoma under androgen receptor pathway inhibition, posing a significant clinical challenge. In a recent landmark study, Wang et al*.* introduced an innovative internal Z-score based approach to comprehensively characterize the transcription factor (TF) landscape in prostate cancer progression, uncovering distinct TF profiles associated with adenocarcinoma and NEPC lineages. Notably, the study proposes a three-phase model of NEPC transdifferentiation—comprising de-differentiation, dormancy, and re-differentiation—revealing dynamic shifts in TF expression that underpin lineage plasticity and therapeutic resistance. This commentary critically evaluates the methodological advancements, the functional significance of the identified TF signatures, and the broader implications of these findings for developing novel therapeutic strategies. By delineating the molecular events driving the transition from androgen receptor (AR)-dependent adenocarcinoma to treatment-resistant NEPC, this work underscores the potential of targeting early and dormant phases of transdifferentiation to improve patient outcomes.

## Introduction

Prostate cancer (PCa) is among the most prevalent malignancies in men and a leading cause of cancer mortality. Advanced therapies targeting the androgen receptor (AR) signaling axis, such as AR pathway inhibitors (ARPIs), have improved outcomes but also led to the rise of treatment-induced neuroendocrine prostate cancer (NEPC), an aggressive, ARPI-resistant variant accounting for ~ 10–17% of cases post-therapy. The emergence of NEPC via transdifferentiation of prostatic adenocarcinoma under therapeutic pressure represents a formidable clinical challenge due to its rapid progression and poor response to standard treatments. A deeper understanding of the molecular underpinnings of NEPC transdifferentiation is thus crucial for developing new interventions [1]. Transcription factors (TFs), as master regulators of cell identity, are suspected to drive the lineage plasticity enabling adenocarcinoma cells to escape AR dependence and acquire neuroendocrine features [2]. However, prior research on TF involvement in NEPC progression has largely centered on a few known factors (e.g. AR, ETS-family members in adenocarcinoma; ASCL1, BRN2/POU3F2, FOXA2, ONECUT2 in NEPC), leaving the broader TF landscape insufficiently explored [3–5]. The study by wang et al. [6] in *Advanced Science* addresses this knowledge gap by comprehensively mapping TF expression profiles across prostate adenocarcinoma and NEPC lineages and investigating their dynamic changes during NEPC transdifferentiation [6]. In this commentary, we critically examine the significance of Wang et al*.’s* findings in the context of prostate cancer research, focusing on how their novel approach illuminates the process of therapy-induced NEPC transdifferentiation and its implications for combating this lethal phenotype.

### Summary of key contributions

The study by Wang et al*.* offers significant insights into the progression of prostate adenocarcinoma (PRAD) to neuroendocrine prostate cancer (NEPC), with a primary focus on a novel three-phase hypothesis of NEPC evolution. First, an internal Z-score algorithm was developed to quantify transcription factor (TF) expression within individual samples, enabling the identification of lineage-specific TF signatures. This analysis revealed 46 adenocarcinoma-enriched TFs (AD-TFs), 56 NEPC-enriched TFs (NE-TFs), and 126 shared TFs expressed in both lineages, with these findings validated across multiple patient cohorts and experimental models. Additionally, functional validation using shRNA-mediated knockdown of AD-TFs and NE-TFs in cell models demonstrated that their depletion significantly impairs proliferation, with AD-TF depletion also reducing migration in adenocarcinoma cells, highlighting these TFs as potential therapeutic targets. Second, the authors utilized a unique patient-derived xenograft (PDX) model (LTL331 → LTL331R) that transitions from adenocarcinoma to NEPC under castration. This enabled a longitudinal analysis of transcription factor dynamics, which revealed coordinated, stage-specific expression programs rather than random transcriptional fluctuations. Based on these patterns, Wang et al*.* propose a compelling “three-phase hypothesis” of NEPC progression – (i) an initial de-differentiation phase, where cells lose adenocarcinoma characteristics; (ii) a prolonged dormancy phase, where cells survive in a suppressed state; and (iii) a re-differentiation phase, where cells acquire neuroendocrine features. As part of this phase, dormant TFs were identified as highly expressed during the transition, offering insights into the TF program in entering and maintain dormancy. Collectively, these findings provide a conceptual breakthrough, offering a mechanistic understanding of how AR-dependent tumors adapt to therapy and evolve into treatment-resistant NEPC. The study lays the foundation for new strategies aimed at targeting lineage reprogramming and tumor dormancy to improve patient outcomes.

### Innovative methodology surpassing traditional analyses

A central strength of this study lies in its methodological approach to identifying key TFs, which addresses the limitations of traditional gene expression analyses. Conventional differential expression methods, such as simple fold-change comparisons between adenocarcinoma and NEPC, can be misleading—often highlighting genes with large fold-changes but low absolute expression while overlooking consistently high yet non-variable TFs. Similarly, standard Z-score normalization across samples tends to obscure biologically meaningful differences within individual tumors and remains highly sensitive to outliers. To overcome these challenges, the study implements an internal Z-score calculation for each TF within individual samples, ranking TFs relative to all others within the same tumor. This within-sample weighting captures the intrinsic prominence of a TF within a tumor’s transcriptome before conducting cross-cohort comparisons. This approach preserves biologically meaningful patterns, such as TFs that consistently rank high across NEPC tumors regardless of absolute expression levels, and it reduces the influence of outlier values. The integration of these rankings across large datasets enables a more precise identification of TFs that distinguish adenocarcinoma from NEPC lineages. Notably, this strategy facilitated the discovery of numerous TFs that conventional analyses had previously overlooked. Prior high-throughput studies in prostate cancer have often relied on techniques such as ATAC-seq or ChIP-seq to map chromatin landscapes and infer TF activity, or have focused primarily on well-characterized transcriptional drivers. While powerful, these approaches are constrained by factors such as antibody availability, sequencing depth, and the inherent limitation that DNA-binding occupancy does not always correlate with functional activity. In contrast, the expression-centric ranking employed in this study offers a complementary perspective, highlighting TFs that emerge as prominent within tumor transcriptomes independently of prior biases. Moreover, this method leverages ubiquitous RNA-seq data, making it broadly applicable with a low technical barrier across diverse datasets and tumor types. By integrating internal normalization with stringent cross-dataset validation, the study extends beyond the well-characterized TFs, uncovering numerous previously unrecognized regulators that likely play critical roles in lineage plasticity. This methodological innovation represents a substantial advancement over traditional comparative analyses, offering a more nuanced and dynamic view of TF regulatory networks in prostate cancer progression.

### Distinct TF landscapes in adenocarcinoma vs NEPC

This study delineates a clear divergence in TF profiles between prostatic adenocarcinoma and NEPC, revealing a broad transcriptional rewiring rather than a switch driven by a few master regulators. Among the 1,639 TFs encoded in the human genome, 46 were identified as AD-TFs and 56 as NE-TFs, with 126 TFs exhibiting high expression in both lineages. The AD-TF set includes well-established luminal-lineage drivers such as AR, NKX3-1, HOXB13, GATA2, MYC, and SPDEF, while the NE-TF set features recognized neuroendocrine-associated regulators like ASCL1, HES6, INSM1, NKX2-1 (TTF1), SOX2, E2F1, FOXA2, FOXM1, and DOT1L. The presence of these lineage “anchor” TFs validates the approach, confirming its ability to capture known biology. Crucially, the study extends beyond these well-characterized TFs, identifying 20 novel AD-TFs and 32 novel NE-TFs. Factors such as ZNF613/614/615, CREB3L1, and THRB are not part of the canonical AR pathway narrative, yet their high expression in AR-driven tumors suggests they may be important cofactors or downstream effectors sustaining the adenocarcinoma state. While NEPC-specific list uncovered TFs like CIC, DLX5/6, HSF2, MAF, SALL2, SATB1, ST18 and others that extend the roster of candidate drivers for NEPC. Notably, some of these (e.g. TEAD1, NKX2-2, HOXB7) had been hinted at in prior studies of NEPC but never deeply investigated, whereas others are entirely new to this context. By expanding the catalog of TFs associated with each prostate cancer lineage, the study opens multiple new avenues for research. Are these novel TFs critical nodes in the regulatory circuitry of the cancer cell, and can they be exploited therapeutically? Functional perturbation experiments confirm the relevance of these TFs, showing that silencing AD-TFs (e.g., NFIX) impairs adenocarcinoma cell growth, while knocking down NE-TFs (e.g., HOXB7) reduces proliferation in NEPC models. Gene ontology and pathway analyses further reveal that while shared TFs govern generic cellular processes, lineage-specific TFs are enriched in developmental and cell fate pathways, reinforcing the concept that NEPC arises through transcriptional reprogramming.

### Dynamic TF expression and the three-phase NEPC transdifferentiation model

The most novel insight from this study is the temporal framework for NEPC transdifferentiation. By analyzing TF dynamics in tumors before, during, and after NEPC emergence in the LTL331/331R PDX model, a non-linear, stepwise trajectory was identified, leading to the “three-phase model” of adenocarcinoma-to-NEPC transition. This model proposes that tumor cells do not switch directly from an adenocarcinoma to a neuroendocrine phenotype but instead undergo a sequential process consisting of de-differentiation, dormancy, and re-differentiation under therapy pressure. In the de-differentiation phase, the majority of AD-TFs exhibit a sharp decline within eight weeks of castration and remain low, reflecting the rapid collapse of the luminal transcriptional program. The dormancy phase follows, characterized by a prolonged period of minimal residual disease, during which both AD-TFs and NE-TFs remain suppressed, evade therapeutic targeting by remaining in a quiescent state. Consistent with this, dormant tumors display low Ki-67 proliferation index and reduced AR and PSA expression, reinforcing the concept of dormancy as a survival mechanism [7, 8]. The re-differentiation phase marks the final transition to NEPC. At tumor relapse, NE-TFs are sharply upregulated, while AD-TFs remain suppressed, signifying the acquisition of a fully neuroendocrine phenotype. Notably, NE-TF activation occurs only after the prolonged dormancy phase, reinforcing the indirect nature of the transdifferentiation process. Instead of a direct lineage switch, the data support a stepwise trajectory in which tumor cells first enter a lineage-negative dormant state before committing to neuroendocrine differentiation. These challenges simplified models of transdifferentiation and provides evidence against alternative hypotheses, such as an epithelial-to-immune-like transition (EIT).

The temporal emergence of several novel TFs during distinct stages of NE transdifferentiation also offers important clues about their potential regulatory functions. For instance, AD-TFs such as NFIX, HOXB13 and GATA2, which show transient upregulation early after androgen deprivation in de-differentiation phase, may be involved in lineage destabilization and facilitating adaptation rather than enforcing lineage identity. During the dormancy phase, the selective activation of dormant-TFs like REST, STAT3, FOXP1, and FOXO3, chromatin remodeling, cellular quiescence, and stem-like properties. Meanwhile, the delayed upregulation of NE-TFs such as CIC, DLX5, and HOXB7, which are mostly absent until the re-differentiation stage, supports their involvement in re-establishing neuroendocrine identity. Although many of these TFs are not yet mechanistically characterized in NEPC, their phased expression trajectory, combined with enrichment in chromatin and developmental pathways, enables a preliminary functional inference. Such timing-based insights help prioritize candidates for further investigation, particularly as potential regulators of therapy-induced plasticity or late-stage NE lineage commitment.

The implications of this three-phase model are substantial. The findings suggest that therapy-resistant NEPC cells—the ones driving relapse—originate from a dormant, minimal residual disease state that remains undetected by standard treatments targeting proliferative or AR-dependent populations. By identifying key transcriptional regulators of dormancy and subsequent reactivation, this study provides potential molecular targets to disrupt the transition to NEPC before relapse occurs. More broadly, these findings refine the understanding of tumor cell plasticity under therapeutic pressure and highlight temporal dynamics in lineage switching as a key vulnerability in treatment-resistant prostate cancer.

### From insights to future directions: transcriptional dynamics, therapeutic implications, and research avenues in prostate cancer

The findings from this study carry several important implications for advancing prostate cancer therapy, particularly for NEPC. The identification of lineage-specific TFs essential for tumor cell survival suggests novel therapeutic targets beyond the AR. Silencing multiple AD-TFs or NE-TFs impairs cell viability, indicating that these tumors depend on their lineage-defining TF networks. The presented TF landscape in the study also underscores the need for personalized and combination therapy approaches. Distinct subsets of AD-TFs and NE-TFs appear to define molecularly separable tumor states, suggesting that individualized treatment based on TF expression signatures could improve precision in therapy selection as well as guidance for combination strategies, which may provide greater efficacy and minimize the risk of clonal escape. Furthermore, the TF signature may also serve as valuable biomarkers. Detecting specific TF signatures in tumor biopsies could alert clinicians to emerging NEPC lineage or dormancy, with a high NE-TF score in castration-resistant tumors potentially signaling incipient neuroendocrine differentiation.

Furthermore, the three-phase model proposed in this study offers a novel framework for implementing longitudinal therapeutic strategies. We suggest a temporal combination approach that targets “gatekeeper” TFs in each phase of NEPC transdifferentiation. During the de-differentiation phase, transiently elevated TFs such as NFIX and HOXB13 may represent early stress responders whose inhibition could limit survival and plasticity under AR suppression. Early intervention during this phase, potentially through combination treatments pairing ARPIs with agents targeting stress-response or epigenetic reprogramming events, might reduce the pool of cells capable of entering dormancy and forestall the emergence of NEPC clones. In the dormancy phase, TFs such as FOXP1, and FOXO3, which implicated in chromatin repression and stemness, might serve as actionable nodes to render them more vulnerable to treatment, or maintain them in a permanently benign state, thereby preventing NEPC relapse. At the re-differentiation stage, reactivated NE-TFs like E2F1, PROX1, ASCL1 and SOX2 become dominant [9, 10], and their blockade may suppress NEPC outgrowth. This phased, TF-guided intervention concept offers a mechanistically informed strategy to delay or prevent NEPC emergence and may complement current androgen-targeted therapies.

In addition to therapeutic implications, the distinct TF signatures uncovered in this study have significant potential for improving disease monitoring and diagnosis. Developing molecular panels based on these signatures could enhance the profiling of tumor samples, especially in heterogeneous metastatic biopsies. Tracking specific TFs in circulating tumor cells or cell-free RNA could enable early detection of neuroendocrine transdifferentiation in patients receiving ARPIs. This would allow clinicians to adjust treatment plans before full NEPC emergence.

Looking forward, several research avenues emerge from these insights. An immediate priority is the functional validation of the novel TFs identified during the transdifferentiation process. Genome editing or overexpression studies could determine whether modulating these TFs drives shifts between adenocarcinoma and NEPC phenotypes, establishing causal roles in lineage switching. In vivo studies utilizing genetic manipulation or small-molecule inhibitors will be critical for assessing the therapeutic potential of these targets. Additionally, exploring the interplay between genetic alterations—such as losses of RB1 or TP53—and TF networks may reveal context-specific dependencies that refine treatment strategies further.

Integrating multi-omics approaches will also be essential for advancing this field. While Wang et al.’s internal Z-score strategy offers a novel lens through which to assess TF activity from bulk RNA-seq data, bulk analysis inherently masks intratumoral heterogeneity by averaging gene expression across diverse cell populations. This limitation is particularly salient in prostate cancer, where rare subpopulations—such as lineage-intermediate or dormant progenitor-like cells—may drive progression toward NEPC. Incorporating single-cell RNA sequencing (scRNA-seq) would enable the resolution of transcriptional programs at the level of individual tumor cells, thereby facilitating the identification of transitional or rare TF-expressing subpopulations that may be obscured in bulk datasets. Such single-cell analyses could refine the three-phase model by delineating trajectories of lineage transition, validating TF co-expression modules, and identifying minority cell types with high plasticity potential. Moreover, combining the internal Z-score approach with chromatin accessibility profiling (e.g., ATAC-seq) or TF motif activity analysis could reveal TFs that are not only highly expressed but also functionally active. As tools for spatial transcriptomics, single-cell multi-omics, and TF-target inference continue to mature, the next phase of research will be well positioned to move beyond static snapshots and toward dynamic, predictive models of prostate cancer lineage plasticity.

In summary, the comprehensive mapping of TF landscapes presented in this study refines the understanding of prostate cancer cell plasticity under therapeutic pressure. The insights gained not only reveal novel therapeutic targets and diagnostic biomarkers but also pave the way for future research aimed at bridging molecular discoveries to clinical impact, ultimately advancing the management of treatment-resistant prostate cancer.

## Data Availability

Not applicable.
